# Investigating the Human Brainstem with Structural and Functional MRI

**DOI:** 10.3389/fnhum.2014.00116

**Published:** 2014-02-28

**Authors:** Florian Beissner, Simon Baudrexel

**Affiliations:** ^1^Department of Neuroradiology, Somatosensory and Autonomic Therapy Research, Hannover Medical School, Hannover, Germany; ^2^Martinos Center for Biomedical Imaging, Department of Radiology, Massachusetts General Hospital, Charlestown, MA, USA; ^3^Department of Neurology, Goethe University Frankfurt, University Hospital, Frankfurt am Main, Germany

**Keywords:** brainstem, fMRI, MRI, physiological noise, autonomic nervous system, neuromodulatory systems, pain modulation, reticular formation

The brainstem is one of the least understood parts of the human brain despite its prime importance for the maintenance of basic vital functions. Owing to its role as a relay station between spinal cord, cerebellum, and neocortex, the brainstem contains vital nodes of all functional systems in the central nervous system, including the visual, auditory, gustatory, vestibular, somatic, and visceral senses, and the somatomotor as well as autonomic nervous systems. The brainstem also contains cholinergic, dopaminergic, noradrenergic, and serotonergic nuclei whose cortical and subcortical projections are essential to the regulation of arousal, behavior, and cognition. Despite this indisputable importance, the brainstem is still largely neglected in attempts to measure or model brain function, especially in human neuroscience. One reason for this neglect is that the anatomical characteristics of the brainstem, specifically its close vicinity to large arteries and ventricles, and the small size of its anatomical substructures, present inherent challenges to neuroimaging analysis. These properties make the brainstem a difficult structure to study with non-invasive methods like magnetic resonance imaging (MRI), as they place high demands on image acquisition as well as data analysis methods. Nevertheless, the field of brainstem-(f)MRI has significantly advanced in the past few years, largely due to the development of several new tools that facilitate studying this critical part of the human brain.

Within this scope, the current goal of this research topic is to compile work representing the state of the art in functional and structural MRI of the human brainstem. We have assembled articles from a number of scientists who have made important contributions to this evolving field, and continue to shape it. The articles have been divided into a functional (Brooks et al., 2013; Henderson and Macefield, 2013; Ress and Chandrasekaran, 2013; Ritter et al., 2013) and a structural section (Deistung et al., 2013; Ford et al., 2013; Lambert et al., 2013; Yeo et al., 2013; Singleton et al., 2014).

The functional section starts with a review by Brooks et al. (2013) that covers the central problem of physiological noise and presents strategies to suppress it. Ritter et al. (2013) have studied the nociceptive system and show its differential reactions to painful skin heating at different slopes. Ress and Chandrasekaran (2013) use the advantages of ultrahigh magnetic field strengths to study the substructure of the inferior colliculus and its tonotopic organization. The functional section concludes with Henderson and Macefield (2013) who provide a review of their extensive research on somatosensory and autonomic centers in the lower brainstem.

The structural section begins with an article by Deistung et al. (2013) introducing quantitative susceptibility mapping as a new means to boost the identification of anatomical details in structural MRI images. Lambert et al. (2013) use quantitative MRI and tensor based morphometry in a large study sample to characterize aging in the human brainstem. Singleton et al. (2014) apply volumetric methods to demonstrate gray matter changes related to meditation and mindfulness-based intervention. Yeo et al. (2013) use probabilistic fiber tracking on diffusion-weighted images to delineate the ascending reticular activating system. Lastly, by using diffusion tensor imaging at ultra high field strengths, Ford et al. (2013) demonstrate precise tractography results of the human brainstem.

The wealth of methods and applications covered by the authors indicates that functional and structural brainstem-MRI methods have developed to a point where they can be applied to study of a wide range of neuroscientific problems. It is the hope of the editors that the brainstem will soon lose its label of a *terra incognita* and become a region of major interest in the neuroscience community.
